# From prohibition to conditional demedicalization: a clinician-informed policy pathway for cannabis use disorder in Iran

**DOI:** 10.1016/j.hpopen.2026.100185

**Published:** 2026-10-02

**Authors:** Mehdi Sayyah, Hamidreza Namazi

**Affiliations:** a Education Development Center, Jundishapur University of Medical Sciences, Ahvaz, Iran; b Department of Medical Ethics, School of Medicine, Medical Ethics and Historyof Medicine Research Center, Tehran University of Medical Sciences, Tehran, Iran

**Keywords:** Cannabis use disorder, Demedicalization, Drug policy, Iran, Decriminalization, Harm reduction, Qualitative research, Policy analysis, Prohibition, Conditional reform

## Abstract

Global debate on cannabis policy has been dominated by jurisdictions moving toward legalization or regulated markets, leaving prohibition contexts analytically underserved. Iran maintains full criminalization of cannabis alongside rising use among young people, no approved pharmacotherapy for cannabis use disorder (CUD) — although off-label options are sometimes used, no medication is approved for CUD in Iran — and a treatment system that clinicians themselves describe as fragmented. Drawing on the first qualitative study of Iranian psychiatrists' views on the demedicalization of CUD, this commentary translates those findings into a testable policy hypothesis for Iranian health authorities: a phased pilot model of conditional demedicalization. By ‘conditional demedicalization’ we mean a partial and reversible withdrawal of the medical frame from mild-to-moderate CUD, coupled with de-penalization of the person who uses, expansion of psychosocial services, and pre-registered stopping rules that allow the reform to be paused if harms emerge. The model comprises five elements: reframing mild-to-moderate CUD as a psychosocial rather than a primarily biomedical problem while preserving medical pathways for severe and comorbid presentations; incrementally substituting therapeutic-supportive referral for criminal sanction against people who use cannabis, informed by the Portuguese dissuasion experience but adapted to existing Iranian harm-reduction infrastructure; redirecting resources from pharmacological management toward evidence-based psychosocial services; an evidence-based prevention shield for adolescents as a precondition of any reclassification; and an intersectoral governance mechanism with explicit monitoring indicators and pre-registered stopping rules. We argue that, in a prohibition context, conditional demedicalization is best understood not as a retreat from care but as a testable harm-reduction hypothesis that, if pilot data support it, could lower barriers to help-seeking while guarding against premature normalization.

## 1 Introduction

The international literature on cannabis policy has grown rapidly, but its geography is skewed. Most empirical and analytical work concerns jurisdictions that have legalized or formally regulated cannabis — Uruguay, Canada, and a growing number of US states — or European settings experimenting with decriminalization [1], [2]. Far less attention has been paid to the policy options realistically available inside strict prohibition regimes, where legalization is not on the political horizon yet patterns of use are changing. Iran is a paradigmatic case. Cannabis is fully criminalized, its use and possession may attract criminal charges, and recreational use remains heavily stigmatized; at the same time, systematic review evidence indicates rising cannabis use, particularly among young people [3]. In that review, the pooled prevalence of past-12-month cannabis use in 2016–2020 was 4.9% among young males and 0.3% among young females, with a significant increase among young males between 2000 and 2020; in the general population, past-12-month use was 1.3% among males and 0.2% among females, and the prevalence of CUD in national studies rose from 0% in 2001 to 0.5% in 2011 [3]. However, large-scale, nationally representative data stratified by age and sex remain limited, highlighting a data gap that the pilot should address. No approved pharmacotherapy for cannabis use disorder (CUD) exists; treatment is mainly psychosocial. National substance-use treatment policy is marked by specific legal contradictions between law-enforcement statutes (which mandate criminalization) and health-sector guidance (which emphasizes treatment); this fragmentation arises from the institutional separation between the Ministry of Interior (security focus) and the Ministry of Health (health focus), and from the absence of an overarching national addiction strategy, and the absence of a coherent conceptual model of addiction [4].

Within this setting, an unusual policy question arises: rather than asking whether cannabis should be legalized — a question with little immediate traction in Iran — one can ask whether cannabis use disorder should remain framed primarily as a medical disease at all, and what a managed, partial demedicalization would mean for stigma, help-seeking, and prevention. This commentary takes up that question. It builds directly on our recent qualitative study, the first in Iran or the wider Middle East to examine psychiatrists' perspectives on the demedicalization of CUD [5], and translates its findings into a testable pilot hypothesis. Our aim is not to relitigate the qualitative findings but to carry them one step further: from description of professional opinion to a specified, phased, and evaluable policy model.

## 2 Medicalization, demedicalization, and the normalization problem

The conceptual vocabulary of this proposal comes from the sociology of medicalization. Conrad [6] described medicalization as the process by which non-medical problems become defined and treated as medical ones; addiction is among his central examples of a condition relocated from the moral and legal registers into the clinical one. Critics of over-medicalization have argued that expanding diagnostic categories produces overdiagnosis, unnecessary treatment, escalating cost, and — crucially for policy — a displacement of responsibility from social structures onto the individual patient and the health system [7], 8.. Rose's [8] injunction to move ‘beyond medicalisation’ does not deny the reality of suffering; it insists that framing a problem as disease is a political act with distributional consequences, determining which institutions are obliged to respond and which are excused. More recent scholarship [9] has examined how the language of public health can be marshalled to support either medicalization or its critique, depending on which harms are foregrounded — a distinction that matters for how demedicalization is framed.

Demedicalization, however, carries its own well-documented hazard in the drug field: normalization. The normalization thesis, as revisited by Pennay and Measham [10], describes the process by which drug use becomes culturally accommodated, with reduced risk perception and wider initiation. Defenders of the brain-disease model of addiction argue precisely that the medical frame protects access to care and signals treatability 11., [12]. Any serious demedicalization proposal must therefore answer a double objection: that it will erode help-seeking by removing the disease label, and that it will erode prevention by removing the danger label. The model we propose below is built around answering both objections simultaneously, which is why we describe it as conditional rather than categorical demedicalization.

A final conceptual clarification is needed, because it is frequently lost in public debate. We distinguish among four related but distinct moves: (1) demedicalization — withdrawing the disease frame; (2) decriminalization — removing criminal penalties for possession/use; (3) de-penalization — replacing criminal with administrative or civil sanctions; and (4) destigmatization — changing social attitudes and norms. These are analytically separable and can occur independently. In Iran, CUD is already treated largely without medication; what is at stake in demedicalization is the framing of the problem and the institutional architecture built around that framing. Conversely, decriminalization concerns the legal status of the person who uses, not the nosological status of the disorder. Our proposal deliberately couples a partial demedicalization of the disorder with a partial de-penalization of the person and with active destigmatization, because the qualitative evidence suggests that these moves reinforce each other in the Iranian context.

## 3 The empirical basis: study methodology and findings

### 3.1 Underlying qualitative study: methodological details

The model presented here draws from a qualitative study of Iranian psychiatrists' perspectives on demedicalization ([5], Discover Mental Health). The study employed inductive content analysis following Graneheim and Lundman [13] — a qualitative data analysis method distinct from phenomenology, designed to describe manifest and latent content in written or verbal data rather than to capture lived experience. This study employed the following methods:

Study Design & Sampling:

Semi-structured interviews with 16 experienced psychiatrists were conducted. Participants were selected using purposive sampling with maximum variation to ensure diverse perspectives across Iran. Sampling continued until theoretical saturation was achieved—the point at which no new conceptual material emerged from additional interviews. This approach is standard in qualitative research and ensures that the sample is broadly representative of perspectives rather than exhaustive.

Participants:

Sixteen psychiatrists were interviewed (9 female, 7 male; mean age 47.1 years, range 38–55 years). Mean clinical experience in addiction medicine was 13.8 years (range 6–22 years). Fourteen held specialist credentials in psychiatry; two held super-specialist credentials. Participants represented diverse settings across Iran, including university clinics, harm-reduction centers, and private practice.

Data Collection:

Interviews were semi-structured, audio-recorded, and transcribed verbatim in Farsi, then translated to English for analysis. Interviews began with open questions (‘Tell me about your experience with demedicalization of cannabis use disorder’ and ‘What would change if cannabis use disorder were no longer considered primarily a medical problem?’) and progressed to focused probing questions based on participant responses and emerging themes. Participants were assured of confidentiality and the voluntary nature of participation.

Analysis:

Data were analyzed using inductive content analysis following the Graneheim and Lundman [13] approach. Transcripts were read repeatedly for immersion. Meaning units were extracted and condensed while preserving core concepts. Codes were generated inductively (not pre-determined by a codebook). Codes were grouped into subcategories and categories based on similarities and differences. Themes emerged through systematic comparison of categories across the dataset. Member checking was performed: preliminary findings and representative extracts from transcripts were returned to a subset of participants for verification and correction. Peer debriefing occurred throughout analysis, with external discussion of codes and emerging themes.

Ethics & Trustworthiness:

The study was approved by the Ethics Committee of the School of Medicine, Tehran University of Medical Sciences (IR.TUMS.MEDICINE.REC.1403.43) on 4 December 2024. Credibility (internal validity) was ensured through prolonged engagement with data (three-month analysis period), member checking with participants, and detailed documentation of methods. Dependability (reliability) was established through a careful audit trail documenting all analytical decisions and through independent review of a sample of coded extracts. Confirmability (objectivity) was maintained through audio recording of all interviews, verbatim transcription, and transparent reporting of analysis steps. Transferability (external validity) was supported by maximum variation in the sample (age, gender, experience level, practice setting) and detailed description of the Iranian context, allowing readers to assess fit to other settings.

### 3.2 Key findings and mapping to policy elements

Analysis yielded 4 main themes with 11 categories and 21 subcategories. Below, we map each theme directly to the policy elements proposed in Section 4, using direct quotes from participants to ground each mapping:

Theme 1: Advantages of Demedicalization (Categories: Stigma Reduction, Empowerment, Reduced Pharmacotherapy, Attention to Structural Roots)

Psychiatrists identified four key advantages of demedicalization. First, reduced stigma: one participant noted, ‘If we stop calling it a disease, people might stop viewing users as sick or broken,’ reflecting the burden of the disease label. This directly supports Element 4.1 (reclassification as psychosocial). Second, enhanced personal empowerment: multiple participants described cases where removing the disease frame paradoxically strengthened engagement, as individuals felt less trapped in a patient role and more capable of change. Third, reduced unnecessary pharmacotherapy: psychiatrists noted that in their experience, patients on anxiolytics or antidepressants for ‘CUD’ often derived more benefit from counseling alone, and that weaning off medication reduced cost and side effects. Fourth, structural attention: participants emphasized that the psychosocial frame pushes attention toward unemployment, poverty, trauma, and family breakdown — the actual roots of problematic use in their clinical experience — rather than keeping focus on the individual's brain or choices.

Theme 2: Disadvantages of Demedicalization (Categories: Medical Neglect, Normalization Risk)

Psychiatrists also articulated two major risks. First, neglect of genuine medical needs: participants worried that ‘if we say it's not a disease, people with real psychiatric comorbidity — depression, bipolar disorder, psychosis — might not get seen or referred for medical help.’ This directly supports Element 4.1's requirement to preserve a medical pathway for severe and comorbid cases. Second, and most emphasized, the normalization risk: psychiatrists repeatedly stated that ‘if we remove the medical label without simultaneously investing in prevention, young people will think cannabis is safe.’ This directly supports Element 4.4's precondition (prevention must run first).

Theme 3: Social-Cultural Impacts (Categories: Employment and Community Reintegration; Prevention Culture)

Psychiatrists described cases where patients, once freed from the ‘addict’ label or the criminal record, regained employment and community roles. This supports Element 4.2's (diversion and de-penalization) emphasis on reducing barriers to social reintegration. Psychiatrists also argued for a shift in public discourse: away from moralization or medicalization, and toward science-based prevention and evidence-centered treatment — directly supporting Element 4.4.

Theme 4: Policy Recommendations (Categories: Therapeutic-Supportive Approaches; Comprehensive Services; Preventing Normalization)

Psychiatrists explicitly recommended replacing criminal sanction with ‘referral to counseling and support services’ (supporting Element 4.2), developing ‘comprehensive psychological and social services’ including family work and vocational support (supporting Element 4.3), and building ‘a strong prevention program for youth’ as a precondition (supporting Element 4.4). These recommendations, drawn directly from clinician experience, form the core rationale for each policy element.

Key Consensus Findings:

Two overarching findings deserve emphasis. First, psychiatrists showed internal consensus favoring a managed transition rather than a binary switch—preserving medical pathways for severe cases while expanding psychosocial options for mild-to-moderate cases. This ‘and-or’ logic (not ‘or’) reflects professional maturity and directly informs the stratified model in Element 4.1. Second, psychiatrists emphasized the specific burden of prohibition: criminalization already drives concealment, secretive use in unsafe environments, and delayed treatment contact. In this context, demedicalization without de-penalization would leave the harms of criminalization untouched. Conversely, de-penalization without prevention and quality services would fail. This led to the sequencing logic in Element 4.5: prevention first, then diversion and services together, then reclassification.

## 4 A five-element model of conditional demedicalization

Importantly, the five-element model presented here is derived from clinician perspectives and is explicitly provisional. We do not propose immediate implementation. Prior to any pilot, a structured multi-stakeholder co-design process (Delphi or consensus workshop) must be conducted that includes people with lived experience of cannabis use and recovery, family members, addiction counselors, social workers, primary-care providers, representatives of the judiciary and law enforcement, and relevant civil-society organizations. The governance committee described in Element 4.5 is the minimum formal mechanism; genuine co-ownership of the model by those most affected is a prerequisite for ethical and feasible implementation.

Preamble: This is a Testable Hypothesis

We propose that this model be piloted as a testable hypothesis by the Iranian Ministry of Health and Medical Education, together with the relevant drug-control and welfare authorities. The model's elements are interdependent and deliberately sequenced. This proposal is offered not as settled policy, but as an evaluable hypothesis. The monitoring framework (element 4.5) is designed to let data overrule the proposal if results do not support key assumptions. We acknowledge the evidence base—16 psychiatrists from one professional constituency—and explicitly frame this as a pilot whose success depends on stakeholder engagement beyond clinicians. Section 5 addresses these gaps.

**Distinguishing study findings from policy assumptions.** Throughout this section we distinguish three kinds of content. (a) Study findings: statements attributed to the psychiatrists in our study (Section 3.2) report results of the qualitative analysis. (b) Design choices: the structure and sequencing of the five elements are the authors' interpretation of those findings. (c) Operational parameters: severity cut-points, the possession threshold, provider numbers, cost ranges, targets, and tripwire values are the authors' provisional assumptions; they were not generated by the study and are expected to be revised through co-design and Year-0 baseline measurement. Each element below opens with a brief statement of its evidence basis.

### 4.1 Reclassify mild-to-moderate CUD as a primarily psychosocial problem, preserving the medical pathway for severe and comorbid cases

**Evidence basis:** The need to relieve mild-to-moderate cases of the disease label while preserving a medical pathway for severe and comorbid cases is a study finding (Themes 1 and 2). The screening instruments, cut-points, and routing rules below are the authors' operationalization.

Operational Definitions:

**Use, disorder, and diversion eligibility.** Three populations must be kept distinct. (i) Cannabis use refers to any use; most people who use cannabis do not meet criteria for CUD [14], [15]. (ii) CUD is a clinical diagnosis made against DSM-5 criteria and graded by severity (below); Element 4.1 applies only to this group, whether or not the person has had contact with the criminal-justice system. (iii) Eligibility for the diversion pathway (Element 4.2) is a legal, not a clinical, category: it is defined by possession for personal use below the threshold and the absence of a supply offence, irrespective of diagnosis. Entry into the diversion pathway therefore does not imply a diagnosis of CUD. People who are diverted are offered voluntary screening; only those in whom CUD is confirmed enter the severity-based service tiers, while those without CUD are offered brief advice and information only.

The dimensional nature of substance use disorders means that not all cannabis use is pathological and that the threshold between recreational use and disorder is fluid [14]. CUD is diagnosed when at least 2 of the 11 DSM-5 criteria are met within a 12-month period; severity is categorized as follows:

• Mild CUD: 2–3 diagnostic criteria met (DSM-5) • Moderate CUD: 4–5 diagnostic criteria met (DSM-5) • Severe CUD: 6 or more diagnostic criteria met (DSM-5)

Screening & Assessment:

Possible CUD will be initially identified using the Cannabis Use Disorders Identification Test–Revised (CUDIT-R; scores ≥8 indicate hazardous use and ≥ 12 possible CUD; [16]) or the WHO Alcohol, Smoking and Substance Involvement Screening Test (ASSIST; cannabis scores of 4–26 indicate moderate risk and ≥ 27 high risk; [17]). Because neither instrument has a validated cut-off that separates mild-to-moderate from severe CUD, screening only identifies who requires assessment; a brief clinical assessment against DSM-5 criteria determines severity and routing. Primary care providers, addiction counselors, or nurse practitioners (not requiring psychiatric referral) will conduct initial assessment. All assessments will include brief screening for current psychiatric comorbidity (depression, anxiety, psychotic symptoms) to ensure appropriate routing.

Service Pathway:

Uncomplicated mild-to-moderate presentations would be managed by default within a psychosocial service tier — counseling, cognitive behavioral therapy, motivational interviewing, family and vocational support, consistent with the psychosocial interventions recommended in international treatment standards [18] — without requiring a psychiatric disease designation as the ticket of entry. Severe CUD (DSM-5 criteria ≥6), presentations with psychiatric comorbidity (major depression, bipolar disorder, psychotic disorders), and all adolescent cases (any age < 18 with CUD diagnosis) would retain a fully medical pathway with psychiatric oversight and potential pharmacotherapy (e.g., for comorbid depression). This directly operationalizes what psychiatrists in our study asked for: relief from the lifelong-patient identity and from unnecessary pharmacological management for the many, without abandoning the medical safety net for the few who need it. It also addresses the brain-disease objection [11]: nothing in this stratification denies the neurobiological reality of severe addiction; it resists only the automatic extension of the disease frame to the entire spectrum of use.

### 4.2 Substitute therapeutic-supportive referral for criminal sanction against people who use cannabis

**Evidence basis:** That fear of legal punishment delays treatment, and that criminal sanction should be replaced by referral to counseling and support, are study findings (Themes 3 and 4). The possession threshold, legal routes, and safeguards below are the authors' proposals.

We acknowledge important structural differences. Portugal's 2001 reform decriminalised all drugs (not cannabis alone), was accompanied by substantial investment in a universal health and social-care system, and operated in a secular legal culture with established independent dissuasion commissions. Iran's legal, religious, and institutional context is markedly different. The Portuguese experience is therefore cited only as a source of design principles (diversion from criminal justice toward health and social support, non-coercive referral, and monitoring) rather than as a model that can be transplanted. Any Iranian pilot would need to adapt these principles to existing harm-reduction centres, the current legal architecture under the Anti-Narcotics Law, and the political constraints described above.

The psychiatrists in our study were unambiguous that fear of legal punishment delays treatment and deepens stigma. We therefore propose that simple use and possession for personal use — operationally defined as possession of cannabis resin or herbal cannabis below a provisional threshold of 5 g — a deliberately conservative figure relative to the reference quantities used under Portugal's ten-day personal-supply rule (5 g for cannabis resin and 25 g for herbal cannabis; [1], 19.) — be progressively routed out of the criminal-justice pathway and into a therapeutic-supportive referral pathway, while supply offences remain untouched by this reform. This threshold is provisional and should be finalized through consultation with the Judiciary and law enforcement before pilot implementation.

#### 4.2.1 Legal and institutional pathway

Refusal of psychosocial services must not trigger criminal sanction or any other penalty. The individual retains the right to decline referral; the only consequence is that the therapeutic-supportive pathway is not entered. This is essential to prevent net-widening and coerced treatment.

Any reform of this kind in Iran must engage with the existing legal architecture rather than assume it away. Under the Anti-Narcotics Law of 1988 (as amended), possession and use of cannabis are criminal offences, though enforcement in practice varies and is often discretionary. Reform could in principle proceed through three routes: (i) legislative amendment by the Majlis; (ii) administrative guidance from the Ministry of Health and the Judiciary; or (iii) judicial interpretation that distinguishes simple possession from trafficking. Each route carries different political and institutional risks. This commentary does not presume to prescribe which is most feasible in the current political moment, but rather argues that the pilot should be designed to proceed under existing administrative authority for referral and treatment, while the question of formal legislative de-penalization is referred to the relevant legislative and judicial bodies.

Several safeguards are essential for ethical implementation:

Non-coercion: Referral to psychosocial services must be genuinely voluntary; a person who declines must not face criminal sanction as a consequence of refusal. Confidentiality: Data generated through screening, referral assessment, or treatment must be protected by law and must not be accessible to law enforcement or used in criminal proceedings against the individual. Non-discrimination: Access to services must not depend on gender, ethnicity, religion, nationality, sexual orientation, or citizenship status. Services must actively recruit and serve marginalized populations. Protection from arbitrary detention: The pilot must include an explicit protocol for documenting and reviewing any detention that occurs during the pilot period, with an independent oversight mechanism. Expungement: The question of whether existing records for simple possession should be expunged or sealed is beyond the scope of this pilot, but we flag it as a necessary component of any longer-term reform.

Legal compliance with international obligations: Iran is a party to the UN Single Convention on Narcotic Drugs (1961) and the Convention against Illicit Traffic in Narcotic Drugs and Psychotropic Substances (1988). These treaties permit considerable flexibility in how possession for personal use is handled, provided that supply-side offences remain criminalized and that the response emphasizes treatment and harm reduction. The proposed pilot is therefore not in itself incompatible with Iran's treaty obligations, though this is a matter for legal experts to confirm (see [20]).

Political Economy and Institutional Feasibility

A proposal of this kind will encounter resistance from actors whose authority or institutional position is tied to the current framing of cannabis as primarily a security and moral issue. These include elements of law enforcement (whose mandate is enforcement, not treatment), parts of the judiciary (particularly judges with conservative jurisprudential views), and some religious authorities (particularly those who interpret Islamic jurisprudence as requiring severe punishment). Conversely, potential supporters include the Ministry of Health (which bears the burden of treating fragmented cases), parts of the State Welfare Organization, and clinician groups whose daily experience is one of inadequate and under-resourced care. The current political window for comprehensive drug-policy reform in Iran is narrow, and we do not claim otherwise. What we argue is that a pilot framed as a health intervention — rather than as decriminalization per se — may be more feasible than a broad legal reform, because it can proceed under existing administrative authority, because its monitoring framework is designed to be paused if harms emerge, and because it leverages existing infrastructure rather than requiring de novo investment. This is a pragmatic, not an ideological, argument. Success will depend on building a coalition that includes health officials, progressive judiciary, and clinicians, while managing the concerns of security-focused actors through transparent monitoring and explicit stopping rules.

### 4.3 Redirect resources from pharmacological management toward evidence-based psychosocial services

**Evidence basis:** The perceived overuse of anxiolytics and the importance of family and vocational support are study findings (Themes 1 and 4). The reallocation model, provider numbers, cost ranges, and coverage targets are the authors' provisional assumptions.

In Iran, no approved pharmacotherapy for CUD exists; current pharmacological expenditure in the population with CUD largely reflects management of comorbid conditions (depression, anxiety) and, as our psychiatrist participants reported, a degree of unnecessary prescribing of anxiolytics (benzodiazepines, buspirone) with attendant dependence risk, cost burden, and treatment abandonment among lower-income patients.

Proposed reallocation: We propose an explicit budgetary reallocation from pharmacological management to psychosocial capacity-building:

1. Expansion of Cognitive Behavioral Therapy (CBT) & Motivational Interviewing (MI) training: Train 50–100 providers (psychologists, counselors, nurses) in CBT and MI per pilot province over 12 months. Indicative cost (illustrative): 500–800 million rials per province per year. Existing facilities at university clinics can host training. Curricula should include cultural adaptation for Iranian context. 2. Family Counseling Programs: Develop and fund family-based intervention protocols (e.g., family support in treatment, marital counseling, parent psychoeducation). Psychiatrists in our study identified family involvement as critical to sustained recovery. Indicative cost (illustrative): 300–500 million rials per province. 3. Vocational and Job-Skills Programs: Partner with employment ministries and NGOs to develop job-readiness training, apprenticeships, and workplace accommodation for people in recovery. Psychiatrists identified vocational reintegration and job skills as among the strongest protections against relapse. Indicative cost (illustrative): 400–700 million rials per province. 4. Psychosocial Service Coverage & Wait Times: Establish baseline and targets. Baseline (to be measured in Year 0): [current % of CUD patients with access to psychosocial services; current mean wait time from referral to first appointment]. Provisional target after pilot Year 2 (authors' assumption; see Section 4.5): coverage ≥60%; wait time < 14 days. Monitor monthly through clinic administrative data.

**Note on cost figures.** The rial amounts above are illustrative, order-of-magnitude figures intended only to indicate the relative scale of the three investment components; they are not the product of a formal costing exercise. Given high inflation and exchange-rate volatility in Iran, nominal figures will date quickly. A formal costing based on local unit costs (trainer and supervisor time, provider stipends, materials, venues, and evaluation) should be undertaken during the Year-0 design phase, before any budget is committed.

This reallocation ensures that a reclassification is backed by material capacity. Without funded psychosocial services, demedicalization would deserve the criticism that it is austerity in disguise. This element is what distinguishes a harm-reduction reform from a disinvestment.

### 4.4 Deploy an evidence-based prevention shield for adolescents as a precondition of reclassification

**Evidence basis:** The warning that removing the medical label may be read by young people as a safety signal, and the call for prevention to come first, are study findings (Themes 2–4). The curriculum content, evaluation metrics, and tripwire values are the authors' proposals, informed by the cited literature.

The single most consistent warning in our qualitative data was that removing the medical label could be read by young people as a safety signal. The normalization literature gives that warning empirical standing 10., 21.. We therefore treat prevention not as an accompaniment to reform but as its logical precondition: an age-appropriate school- and media-based education program must be operating and demonstrating no adverse trends before any public reclassification is announced.

Prevention Program Specifications:

Target Population: Adolescents ages 13–18 (middle and high school) Delivery: In-school curriculum + social media and radio campaigns in Farsi + optional parent psychoeducation workshops Curriculum content (9–10 h annually, integrated into health classes): 1. Prevalence data for cannabis use in Iran by age and trend (national and regional; [3]) 2. Neurobiological effects on adolescent brain development (focus on prefrontal cortex maturation, decision-making, impulse control, reward sensitivity; [21]) 3. Cannabis-psychosis vulnerability: Honest, evidence-based communication. Avoid exaggeration (which prior Iranian education efforts used, and which youth now dismiss). State: ‘Regular cannabis use in adolescence, especially in people with family history of psychotic illness, is associated with increased risk of psychotic symptoms. This is not inevitable, but the risk is real.’ This frames risk as conditional and developmental, not deterministic [14]. 4. Occupational and educational impairment: Evidence for grade decline, school dropout, work performance effects (quantified, age-appropriate) 5. Addiction potential: Clarify that approximately 9% of people who use cannabis develop addiction, rising to about 17% among those who begin use in adolescence [15] 6. Social and relational impacts: Changes in peer relationships, family conflict, driving safety Framing principle: Scientific accuracy over fear appeals. Psychiatrists stressed that exaggerated messaging discredits education; credible risk communication can displace myth. Evaluation metrics (pre/post-test; annual cohort follow-up): • Knowledge gain: Pre/post-test improvement ≥30% in correct answers • Risk perception: Validated scales (e.g., perceived-harm scale) — target: no decline from baseline • Behavioral intent: ‘If offered cannabis, would you try it?’ — target: no increase in affirmative responses post-intervention Timeline: Prevention program must be designed, piloted in schools, and running for ≥6 months with baseline data collected before Element 4.1 (reclassification) is announced publicly. Tripwire Thresholds (pre-registered, objective triggers): • Adolescent past-year prevalence (age 15–17): Baseline [national school survey estimate, to be specified]. If increase ≥5 percentage points in Year 1 or Year 2 → reform pauses pending review of prevention program • Age of first use: Baseline [median age, to be measured]. If declines ≥1 year → review triggered • Risk perception (validated scale): If declines >15 percentage points → pause reclassification announcement and intensify prevention.

### 4.5 Establish intersectoral governance with explicit monitoring indicators and pre-registered stopping rules

**Evidence basis:** The preference for a managed, sequenced transition is a study finding (Key Consensus Findings). The committee composition, indicators, targets, and stopping rules are the authors' proposals and have not yet been tested.

Governance Committee Composition:

• Ministry of Health (chair) — 1 representative • State Welfare Organization — 1 representative • Ministry of Education — 1 representative • Judiciary — 1 representative (judge or legal advisor) • Law Enforcement / Ministry of Interior — 1 representative • Civil society: People with lived experience of cannabis use — 1 representative • Civil society: Family members / caregivers — 1 representative Total: 7 members. Committee meets quarterly minimum. Decisions made by consensus where possible; disputes escalated to Ministry of Health for resolution. Authority & Budget: • Standing budget: to be determined in the Year-0 costing exercise (see Section 4.3) for administration, data collection, external evaluation, and committee operations • Accountability: Quarterly progress reports to provincial health authority; annual public summary report with data dashboard • Transparency: All indicators, stopping rules, and baseline values published at outset; public dashboard updated quarterly; annual external evaluation report (independent academic team).

All baseline values marked “to be measured” will be established in a dedicated Year-0 data-collection phase using existing Iranian sources where available (e.g., [3] for adolescent prevalence; [22] for adult drug use disorder prevalence and service utilization; national treatment registry data for treatment-seeking; police and prosecutor statistics for diversion). Where national data are currently incomplete, the pilot protocol will include a rapid baseline survey before any service or legal changes begin. Stopping rules will be pre-registered with the Ministry of Health and an independent evaluation team before Year 1.

**Justification of numerical thresholds.** The targets and tripwire values in Section 4.4 and Table 1 (e.g., a ≥ 5 percentage-point rise in adolescent past-year prevalence, a > 15 percentage-point decline in risk perception, a ≥ 1-year fall in age of first use, a ≥ 30% knowledge gain, psychosocial coverage ≥60%, diversion ≥50%, and treatment-seeking ≥20%) are the authors' provisional, pre-specified decision thresholds. They were not generated by the qualitative study and are not derived from a formal statistical model. They were chosen to be large enough to exceed plausible year-to-year sampling variation in school and population surveys, yet small enough to trigger review before harms become entrenched; coverage and reach indicators follow the implementation-outcomes framework of Proctor et al. [23]. All values should be recalibrated once Year-0 baselines and their confidence intervals are available — for example, by expressing the prevalence tripwire relative to the measured baseline — and then fixed in the pre-registered protocol.

**Table 1:** Monitoring indicators, baselines, targets, and decision rules.

Indicator	Data source	Frequency	Baseline & target	Decision rule
Treatment-seeking rate (% of estimated CUD population initiating contact annually)	National CUD registry + pilot clinics	Quarterly	To be measured Year 0 Target Year 2: ≥20%	If <10% after Year 2 → Reassess service capacity & funding
Adolescent prevalence (past-year use, ages 15–17)	School-based national survey	Annual	Baseline [from [3] or new survey] Target: No increase	If ≥5 pp. increase in Year 1–2 → Pause reform pending review
Criminal-justice diversion rate (% of cannabis contacts diverted from prosecution)	Police & prosecutor records	Quarterly	Baseline: 0% Target Year 2: ≥50%	If <20% by Year 2 → Consult Judiciary on enforcement authority
Psychosocial service coverage (% of identified cases offered services)	Pilot service records	Quarterly	To be measured Year 0 Target: ≥60%	If <30% by Year 2 → Reassess funding & training
Risk perception (validated scale; no decline)	Annual population survey	Annual	Baseline Year 0 Target: No decline from baseline	If ↓ >15 pp. → Pause reclassification; intensify prevention

## 5 Anticipating objections and addressing gaps

### 5.1 Disease model and access to care

**Claim:** Defenders of the disease model argue that any softening of the medical frame reduces access to care 11., [12].

**Reply:** In a prohibition context, the empirical bottleneck to care is not an insufficiently medical framing but fear of legal exposure and stigma. The model expands, not contracts, the routes into support. First, it preserves the medical pathway precisely where the disease model is strongest—severe and comorbid presentations. Second, it creates an alternative (non-stigmatizing) pathway for mild-to-moderate CUD, thereby broadening access. Third, diversion (element 4.2) removes the legal barrier that currently deters help-seeking. Evidence from Portugal is consistent with this logic: after the 2001 reform, which paired decriminalization with expanded treatment services, uptake of drug treatment increased, although attributing this change to decriminalization alone is contested [1].

Critical untested assumption: Dual-outcome separation. The model's success depends on separating stigma reduction from risk perception in practice. No comparative evidence from another jurisdiction currently demonstrates that this has occurred. This is a hypothesis we propose to test, not a proven mechanism. If monitoring data show that stigma reduction correlates with risk perception decline >15 percentage points, the reform should pause and be redesigned. This is precisely why the stopping rule in Table 1 exists.

### 5.2 Is this demedicalization, or simply stratified care?

A fair objection is that what we propose is not demedicalization at all, but rather a stratified, rights-based public health model that preserves medical pathways for severe cases and expands psychosocial services for mild-to-moderate cases. We accept this framing. The term ‘conditional demedicalization’ is used here because it captures the specific move we are proposing — a partial withdrawal of the medical frame — but we do not insist on the terminological choice. If ‘stratified care’ or ‘public health-oriented reclassification’ communicates the proposal more accurately to Iranian policymakers and clinicians, we would welcome that reframing. The substance is what matters: mild-to-moderate cases are managed without the disease label; severe and comorbid cases retain full medical care; the person is de-penalized; services are available; prevention is protected; data decide.

### 5.3 Does this individualize responsibility?

There is a risk that framing mild-to-moderate CUD as a ‘psychosocial problem’ shifts responsibility from structural determinants — unemployment, poverty, trauma, social exclusion — onto the individual. We explicitly reject this reading. The psychosocial tier must include, not replace, structural interventions: Element 4.3 specifies vocational programs, housing support (in coordination with welfare agencies), family services, and community-level prevention as integral components. Individual counseling alone will not address the conditions that produce problematic use. Psychiatrists in our study repeatedly emphasized this point: the move from medicalization is not toward individual blame, but toward social and structural attention.

### 5.4 What about supply-side enforcement?

The proposal deliberately leaves supply-side enforcement (trafficking, large-scale production) untouched. This is a pragmatic choice, not a normative commitment. We acknowledge that without reform of supply-side enforcement, black-market harms will persist, and that this limits what a demand-side pilot can achieve. Supply-side reform is a larger and politically more fraught question, involving coordination with international drug-control treaties, law enforcement culture, and organized-crime dynamics. A demand-side pilot can proceed independently and can demonstrate (or fail to demonstrate) benefits that may later inform supply-side reform. This is a staged-reform logic, not an evasion.

### 5.5 From a single study to a consensus process

We acknowledge that this model derives from a single qualitative study of 16 psychiatrists — one professional constituency with its own institutional stakes in the medicalization debate. This is a limitation, not a foundation for policy. We therefore recommend that the next step should not be immediate implementation, but rather a structured consensus and co-design process: a Delphi study or multi-stakeholder consultation involving (in addition to psychiatrists) addiction counselors, social workers, people with lived experience of cannabis use and recovery, family members, law enforcement, judiciary, policymakers, and religious scholars. The five-element model presented here should be treated as a starting point for that process, not its conclusion. A refined logic model mapping each element to objectives, activities, outputs, outcomes, indicators, assumptions, and risks (see Supplementary Table 2) would be a natural output of that consensus process.

## 6 Equity, ethics, and lived experience

Representation of people with lived experience on the governance committee is necessary but not sufficient. Their meaningful involvement in the prior co-design phase is required so that the model itself is not solely clinician-derived.

The proposal affects people who use cannabis, their families, and communities. Yet lived experience is largely absent from this model's design. This is a critical gap that must be addressed in implementation.

Equity Risks: The reform could inadvertently create a two-tier system where privileged groups access high-quality psychosocial care while marginalized groups remain criminalized or diverted only nominally. Gender-specific risks are particular: women in Iran face compounded legal and social stigma for substance use. To mitigate:

• Ensure psychosocial services are universally accessible (free or subsidized) regardless of employment, insurance status, or ability to pay • Disaggregate monitoring data (Table 1) by sex, age, province, urban/rural setting, ethnic/religious background, and socioeconomic status; report findings by these strata • Develop gender-sensitive service standards and train providers in trauma-informed, culturally competent care • Ensure governance committee representation from marginalized groups (LGBTQ+ individuals, ethnic minorities, rural residents, internally displaced persons) • Include women's organizations and gender-focused NGOs in implementation and monitoring.

Voluntary Participation: Therapeutic-supportive referral must be genuinely voluntary. While courts may order treatment, the content of that treatment — the specific modality, provider, timing — should be negotiated with the individual, not imposed. This protects autonomy and improves engagement. Confidentiality and Protection: Data collected for the pilot must be rigorously protected by law. Information shared in psychosocial counseling must absolutely not be fed back to law enforcement or used in criminal proceedings. If this protection cannot be guaranteed in law, the pilot should not proceed. Confidentiality is not a nice-to-have; it is a prerequisite for ethical reform.

## 7 Conclusion

Prohibition contexts are not policy deserts. Between the status quo of criminalized, stigmatized, and medically framed cannabis use and the distant horizon of legalization lies a tractable middle path: conditional demedicalization, in which the disorder is partially reframed, the person is progressively de-penalized, psychosocial services are funded to receive them, adolescents are shielded by credible prevention, and the whole arrangement is monitored against explicit indicators with the willingness to stop if it fails. Iranian psychiatrists—the professionals closest to this patient population—have sketched this path through their experiences and insights. What we have done here is specify it operationally, ground it in realistic implementation models (the Portuguese analogy, not transplant), and design a monitoring framework that privileges evidence over ideology. We offer this model to Iranian health authorities as a pilot proposal. It is intended to generate evidence, not to settle policy. We call for research that prioritizes the perspectives and involvement of people with lived experience, rigorous evaluation by an independent team, and honest reporting of both successes and failures. To the international drug-policy community, we offer a reminder: prohibition contexts are under-represented in the international cannabis-policy literature, and their experiences may offer useful lessons for comparative policy analysis—though we make no claim that they are uniquely instructive. What we do claim is that reform inside prohibition regimes has its own logic, its own constraints, and its own possibilities. Engaging seriously with those possibilities is an overdue scholarly and practical task.

## CRediT authorship contribution statement

**Mehdi Sayyah:** Resources, Project administration, Methodology, Investigation, Data curation, Conceptualization. **Hamidreza Namazi:** Visualization, Validation, Supervision, Software.

## Ethical approval

This commentary reports no new primary data. The underlying qualitative study received ethical approval from the Ethics Committee of Tehran University of Medical Sciences (Approval Code: IR.TUMS.MEDICINE.REC.1403.43; Approval Date: March 2025). This commentary does not require separate ethical review as it synthesizes and analyzes previously approved qualitative research.

## Ethics

This commentary reports no new data. The underlying qualitative study ([5], Discover Mental Health) was approved by the Ethics Committee of the School of Medicine, Tehran University of Medical Sciences (IR.TUMS.MEDICINE.REC.1403.43) on 4 December 2024.

## Funding

This work received no specific grant from any funding agency in the public, commercial, or not-for-profit sectors. The underlying qualitative study [5] was conducted as part of routine clinical education and professional development activities at Ahvaz Jundishapur University of Medical Sciences and received no dedicated funding.

## Declaration of competing interest

The authors declare that they have no known competing financial interests or personal relationships that could have appeared to influence the work reported in this paper.

## Data Availability

No new data generated in this study. The underlying study data are not publicly available due to confidentiality protections for research participants, but may be available upon request to the corresponding author following institutional review.
