# Supplementary material for: From prohibition to conditional demedicalization: a clinician-informed policy pathway for cannabis use disorder in Iran
Source: Health Policy Open. 2026 Oct 2;11:100185. doi: 10.1016/j.hpopen.2026.100185 (PMC13670646; doi:10.1016/j.hpopen.2026.100185)
Supplement: Supplementary file 1 [file mmc1.docx]

| **Element** | **Objective** | **Key Activities** | **Outputs** | **Outcomes** | **Indicators** | **Assumptions & Risks** |
| --- | --- | --- | --- | --- | --- | --- |
| **4.1 Reclassify mild-to-moderate CUD as psychosocial; preserve medical pathway for severe/comorbid cases** | Reduce over-medicalization of mild-to-moderate CUD while protecting severe and comorbid cases | DSM-5 severity assessment; CUDIT-R (<24) and ASSIST (cannabis <27) screening; PHQ-9, GAD-7, and single-item psychosis screen in primary care; routing protocol to psychosocial vs psychiatric tier | Screening protocol approved; primary-care providers trained; proportion of cases correctly classified | Mild-to-moderate cases managed in psychosocial tier without disease label; severe and comorbid cases retain psychiatric oversight | % correctly classified as mild/moderate vs severe; % referred to psychosocial vs psychiatric tier; rate of missed severe cases | **Assumptions:** providers competent in DSM-5; screening tools validated in Farsi. **Risks:** under-detection of severe or comorbid cases; over-routing to psychosocial tier. **Mitigation:** supervision, DSM-5 confirmation protocols, audit of a sample of classifications |
| **4.2 Substitute therapeutic-supportive referral for criminal sanction (possession ≤5 g)** | Remove legal barrier to help-seeking while leaving supply-side enforcement untouched | Police and judiciary training; diversion protocol; confidentiality law; non-coercion protocol; documentation of any detention during pilot | Diversion protocol approved; officers and judges trained; referral logs established | 50% of eligible possession cases routed to health services instead of prosecution; no penalty for refusal of services | Diversion rate (% of eligible cases); treatment entry rate among diverted individuals; rate of refusal without penalty | **Assumptions:** police compliance; judiciary cooperation; confidentiality enforceable in law. **Risks:** net-widening; coerced treatment; confidentiality breach; police discretion uneven. **Mitigation:** voluntary participation safeguards; legal data protection; independent oversight; explicit non-coercion clause |
| **4.3 Reallocate resources from pharmacological management to psychosocial services** | Build real psychosocial service capacity to receive diverted and reclassified cases | Train 50–100 providers per pilot province in CBT and MI; establish family-based services (family counseling, parent psychoeducation); develop vocational and job-skills programs with employment ministries and NGOs | 50–100 trained providers per province; family service protocols; vocational program partnerships | ≥60% of identified mild-to-moderate cases access psychosocial services; wait time <14 days; ≥50% retention (≥4 sessions) | Service coverage (%); wait time from referral to first session (median days); retention rate (≥4 sessions); disaggregated by sex, age, province, urban/rural | **Assumptions:** budget released (2.0–2.5B rials/province/year); qualified workforce available; training infrastructure exists at university clinics. **Risks:** workforce shortage; low retention; funding not released. **Mitigation:** training pipeline; retention incentives; remote supervision; ring-fenced budget |
| **4.4 Deploy evidence-based adolescent prevention shield as precondition of reclassification** | Maintain adolescent risk perception and prevent normalization before any public reclassification is announced | School-based curriculum (grades 9–12; 6–10 hrs/year); media and radio campaign in Farsi; parent psychoeducation workshops; teacher training; pre/post evaluation; annual cohort follow-up | Curriculum developed and delivered; teachers trained; youth reached; baseline prevalence and risk perception measured | Knowledge gain ≥30% pre/post; no decline in risk perception; no increase in adolescent past-year prevalence; no decline in age of first use | Knowledge gain (%); risk perception (validated scale); adolescent past-year prevalence (ages 15–17); median age of first use | **Assumptions:** teachers trained; messaging consistent across school and media; curriculum culturally adapted. **Risks:** fear-based messaging discredits prevention; education itself normalizes use; inconsistent messaging. **Mitigation:** science-based honest communication; independent evaluation; pre-registered tripwires; process evaluation (fidelity, engagement) |
| **4.5 Establish intersectoral governance with monitoring and pre-registered stopping rules** | Ensure data-driven, reversible decision-making and public accountability | 7-member governance committee (MOH chair; Welfare; Education; Judiciary; Law Enforcement; person with lived experience; family member); centralized de-identified registry; quarterly data review; annual public dashboard; independent external evaluation | Committee established; registry operational; dashboard live; external evaluation team appointed | Public transparency; reversible actions via pre-registered stopping rules; continuity of care if pilot paused | 5 indicators monitored quarterly (treatment-seeking, adolescent prevalence, diversion, service coverage, risk perception); stopping rules triggered or not | **Assumptions:** committee consensus possible; data quality adequate; political will to publish. **Risks:** mission creep; politicization; data misuse; confidentiality breach. **Mitigation:** objective pre-registered stopping rules (not discretionary); independent evaluation; legal data protection; published decision rationale |

**Table 2. Logic model for the five-element conditional demedicalization framework: objectives, activities, outputs, outcomes, indicators, and assumptions/risks.**
